# Epithelial microfilament regulators show regional distribution in mouse conjunctiva

**Published:** 2010-10-31

**Authors:** Hong-Yuan Zhu, A.K. Riau, R.W. Beuerman

**Affiliations:** 1 Singapore Eye Research Institute, Yong Loo Lin School of Medicine, National University of Singapore, Singapore; 2 Department of Ophthalmology, Yong Loo Lin School of Medicine, National University of Singapore, Singapore; 3 Duke-NUS SRP Neuroscience and Behavioral Disorders, Singapore

## Abstract

**Purpose:**

The conjunctival epithelium is a continuous sheet of cells with regional characteristics that appear to be similar. This study was designed to investigate the distribution and levels of expression of a subset of microfilament regulators in the forniceal, palpebral, and bulbar conjunctival epithelia.

**Methods:**

Balb/C mice were used. The localizations of paxillin, focal adhesion kinase, vinculin, talin1, cofilin, profilin, gelsolin, integrin β1, and integrin α6 were studied with the use of cross-sectional immunofluorescent staining. For a detailed cellular analysis, positioning and ablation with the laser microbeam (PALM) Combi System was used to obtain forniceal, bulbar, and palpebral conjunctival epithelia for expression comparison with the use of western blot analysis and quantitative real-time polymerase chain reaction.

**Results:**

Immunostaining showed that focal adhesion kinase, cofilin, profilin, gelsolin, talin1, and vinculin were expressed in all layers of the forniceal, palpebral, and bulbar conjunctival epithelia. Paxillin, integrin β1, and α6 was found to be located in the basal cell layer in all three of these areas. Quantitative real-time polymerase chain reaction showed that the transcript levels of these microfilament regulators in the forniceal conjunctivae were higher than those levels found in the bulbar and palpebral conjunctivae. Western blot analysis confirmed the differential expression levels of these microfilament regulators in the forniceal, bulbar, and palpebral conjunctivae.

**Conclusions:**

Differences in the levels of microfilament regulators in the forniceal, bulbar, and palpebral conjunctivae suggest different modes of interaction with their microenvironment and within cell layers.

## Introduction

The ocular surface is composed of two adjacent epithelia that form the outer layer of the cornea and the conjunctiva. These two epithelia have clearly distinguishable phenotypes that include distinct patterns of expression of tissue-specific cytokeratins (CKs) and separate stem-cell origins [1]. The initial differentiation of the ocular surface epithelia is associated with a switch of CK expression from CK5 and CK14 to the tissue-specific CK3 and CK12 for the cornea and CK4 for the conjunctivae [2,3]. Corneal stem cells are thought to localize to the basal layer of the limbus [4]. Outside the cornea, the conjunctival epithelium is regarded as a continuous sheet of cells without regional specialization. However, conjunctival stem cells have been suggested to be located in more than one area, including the palpebral [5], bulbar [6,7], and forniceal conjunctivae [8,9].

The possibility of more than one conjunctival stem cell niche raises questions about the molecular diversity of these sites. Are conjunctival cells phenotypically similar across these diverse regions? Since CKs can differentiate two cell types (i.e., corneal and conjunctival epithelial cells), the interaction of the intracellular microfilaments with the extracellular microenvironment (EME) may also be important in cell differentiation.

Integrin-mediated adhesion complexes provide both physical and regulatory links between the intracellular microfilament system and the EME [10-14]. Integrin-mediated adhesion complexes include signaling proteins such as focal adhesion kinase (FAK) as well as integrins and microfilament regulators such as talin, vinculin, and paxillin [15]. These microfilament regulators modulate the assembly and disassembly of actin filaments [16], and they act cooperatively to control the precision of events such as cell adhesion, movement, and proliferation [17-19].

This study examined the expression of a subset of microfilament regulators in the forniceal, bulbar, and palpebral conjunctival epithelia of the mouse with the use of real-time polymerase chain reaction (RT–PCR), western blot analysis, and immunofluorescent staining aided by the laser dissection of selected cell layers to decipher the molecular components that mediate the interaction between the intracellular microfilament system and the EME of the conjunctivae at forniceal, palpebral, and bulbar sites.

## Methods

### Animals

In this study, Balb/C mice of both sexes were used in accordance with the ARVO recommendations for animal experimentation. All protocols that involved animal use were approved by the SingHealth IACUC.

### Immunostaining

Conjunctival tissues from the mouse eye (n=8) were embedded in Optimal Cutting Temperature compound (OCT; Leica, Nussloch, Gottigen, Germany). Prepared tissue blocks were sectioned at 10 μm and fixed with acetone at 4 °C for 20 min. After blocking with 5% normal goat serum in 1× phosphate-buffered saline (PBS; 1st Base, Singapore) for 30 min, primary antibodies (Table 1) were applied at the specified dilutions in 5% goat serum and left overnight at 4 °C. After washing with 1× PBS, the appropriate fluorescein-isothiocyanate–conjugated anti-mouse, anti-rat, and anti-rabbit secondary antibodies (1:500; Invitrogen, Carlsbad, CA) were applied in 1× PBS for 1 h in a dark incubation chamber. After washing with 1× PBS, UltraCruz Mounting Medium that contained 4,6-diamidino-2-phenylindole (Santa Cruz Biotechnology, Santa Cruz, CA) was applied. A fluorescence microscope (Zeiss, Oberkochen, Germany) was used to examine the slides and to take photographs. Primary antibodies were omitted for negative controls.

**Table 1:** Antibodies used in immunofluorescence and western blot.

**Target antigen**	**Source/catalog No.**	**Host**	**Working dilution**
Cofilin	NOVUS, Littlton, CO/NB100–81866	Rabbit polyclonal	1:1000 (WB) 1:100 (IF)
Gelsolin	BD Transduction laboratories, Missisauga, CA/610412	Mouse monoclonal	1:2500 (WB) 1:100 (IF)
Profilin-1	Cell Signaling, Danvers, MA/3237	Rabbit polyclonal	1:1000 (WB) 1:100 (IF)
Integrin beta1	Millipore, Temecula, CA/MAB1997	Rat monoclonal	1:1000 (WB) 1:100 (IF)
Paxillin	Abcam, Cambridge, UK/ab32084	Rabbit monoclonal	1:1000 (WB) 1:100 (IF)
Vinculin	Sigma, St. Louis, MO/V9131,	Mouse monoclonal	1:1000 (WB) 1:100 (IF)
Talin	Sigma, St. Louis, MO/T3287	Mouse monoclonal	1:1000 (WB) 1:100 (IF)
FAK	Abcam, Cambridge, UK/ab40794	Rabbit monoclonal	1:1000 (WB) 1:100 (IF)
Integrin alpha6	Millipore, Temecula, CA/MAB1378	Rat monoclonal	1:1000 (WB) 1:100 (IF)
Cytoketatin 4	Acris, Herford, Germany/BM559	Mouse monoclonal	1:100 (IF)
Phalloidin-FITC	Sigma, St. Louis, MO/p5282		1:200 (IF)

In the “Working dilution” column, WB indicates western blot and IF indicates immunofluorescent staining.

### Laser-capture microdissection of conjunctival epithelial cells

Laser-capture microdissection was performed as described previously [20] to obtain full-thickness epithelial samples from forniceal, palpebral, and bulbar conjunctivae. Epithelial cell samples were collected into the caps of 0.5 ml tubes that contained 40 μl of Trizol for RNA extraction or 40 μl of radioimmunoprecipitation assay lysis buffer (RIPA; Santa Cruz Biotechnology) with protease inhibitor for protein extraction.

### Polymerase chain reaction

RNA extraction and the reverse transcription of 100 ng of RNA for each sample were performed as previously described [20]. Table 2 lists the primers that were used to detect the transcripts of the microfilament regulators. RT- PCR was performed with the use of the LightCycler 480 System (Roche Diagnostics, Basel, Switzerland). For each reaction, the appropriate probe was selected from the Universal ProbeLibrary (ProbeFinder web-based assay design tool). Glyceraldehyde-3-phosphate dehydrogenase (*GAPDH*) was used as the internal control. mRNA was obtained from three independent experiments via the use of three experimental groups with 20 mice in each group (n=60). Negative controls included H_2_O and a mixture of the reverse transcription reaction without reverse transcriptase. A nontemplate control was included to detect DNA contamination. The conjunctival forniceal epithelial sample was used as the calibrator for comparing the relative abundance of each target gene in the palpebral and bulbar conjunctivae samples. Delta Ct (ΔCt) was calculated by subtracting the Ct of *GAPDH* from the Ct of the targeted gene. The fold change was determined with the use of the following equation:

**Table 2:** Primers used in quantitative real-time PCR.

**Gene**	**NCBI number**	**Primer sequence**
GAPDH	NM_008084.2	Left Primer: TGTCCGTCGTGGATCTGAC
		Right Primer: CCTGCTTCACCACCTTCTTG
Vinculin	NM_009502.4	Left Primer: CCTCAGGAGCCTGACTTCC
		Right Primer: AGCCAGCTCATCAGTTAGTCG
Profilin-1	NM_011072.4	Left Primer: CTGTCACCATGACTGCCAAG
		Right Primer: GATCAAACCACCGTGGACA
FAK	NM_007982.2	Left Primer: CCCCGCTGCCTTCTATCT
		Right Primer: TCCTCTTTACATTGTAGCCCAGA
Gelsolin	NM_146120.3	Left Primer: CAAAGTCGGGTGTCTGAGG
		Right Primer: CTTCCCTGCCTTCAGGAAT
Integrin α6	NM_008397.3	Left Primer: ATTCAGGAGTAGCTTGGTGGAT
		Right Primer: TTCTCTTGAAGAAGCCACACTTC
Integrin β1	NM_010578.2	Left Primer: TGGCAACAATGAAGCTATCG
		Right Primer: ATGTCGGGACCAGTAGGACA
Paxillin	NM_011223.2	Left Primer: GGACTGGCGTCTGAGGAC
		Right Primer: ACACTGGCCGTTTGGAGA
Talin1	NM_011602.5	Left Primer: CTGGCCTCACAAGCCAAG
		Right Primer: TTGATGTGAGCGCCTATCTCT
Cofilin1	NM_007687	Left Primer: TCTGTCTCCCTTTCGTTTCC
		Right Primer: TTGAACACCTTGATGACACCAT

$${\text{2}}^{\left(-\Delta \Delta \text{Ct}\right)}\text{ where }\Delta \Delta \text{Ct }=\;\Delta {\text{Ct}}_{\text{sample}}-\;\Delta {\text{Ct}}_{\text{calibrator}}$$.

### Western blot analysis

For the western blot analysis of microfilament regulators, protein was obtained from three independent experiments of three experimental groups with 20 mice in each group (n=60). The forniceal, palpebral, and bulbar conjunctivae were separately dissected and harvested in RIPA buffer, and the lysates were then analyzed by western blot. Protein concentrations were determined with the use of a bicinchoninic acid protein assay kit (Pierce Biotechnology, Rockford, IL) according to the manufacturer’s instructions. Total lysates (40 μg) were loaded on SDS–PAGE gels, transferred to nitrocellulose paper, and blotted with the primary antibodies specified in Table 1. All antibodies were incubated overnight at 4 °C and blotted with specific horseradish-peroxidase––conjugated secondary antibodies purchased from Santa Cruz Biotechnology (1:2,000 for anti-rabbit antibody sc-2030, 1:2,000 for anti-mouse antibody sc-2005, and 1:5,000 for anti-goat antibody sc-2350). The same membrane was then reprobed with an antibody to GAPDH (Santa Cruz Biotechnology) as an internal control to ensure equal protein loading in all lanes. The membrane was developed with SuperSignal West Pico chemiluminescent substrates (Pierce Biotechnology). The X-ray films (Pierce Biotechnology) were scanned, and the band intensity was quantified by densitometry with the use of Kodak molecular imaging software. The densitometry readings for each protein were first corrected by the corresponding background and then compared with the conjunctival fornix. The fold increase of the conjunctival fornix was set as 1.

### Statistical analysis

Values are expressed as mean±standard deviation. Statistical analysis was performed by one-way ANOVA (Statistica 6.0; SPSS, Chicago, IL) followed by the Tukey post-hoc test. A probability level of p<0.05 was considered to be statistically significant.

## Results

### Immunostaining of microfilament regulators

**Figure 1:**
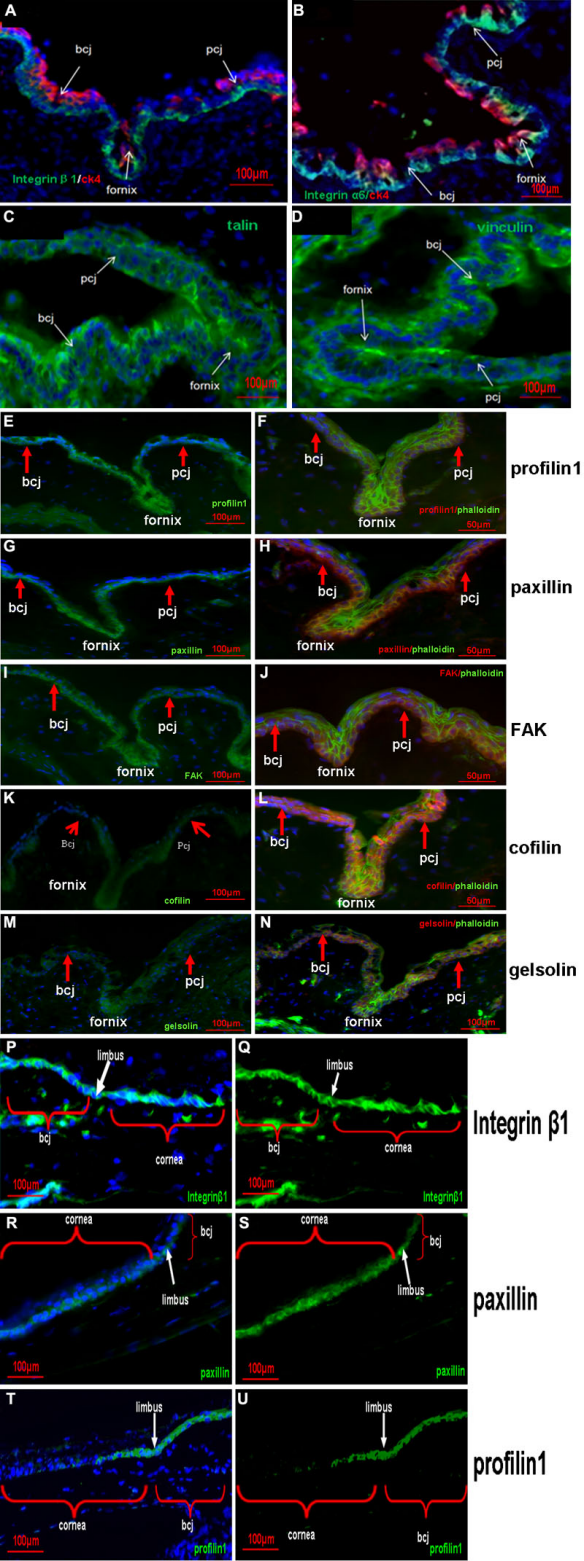
Distribution of microfilament regulators in the conjunctiva. bcj, bulbar conjunctiva; pcj, palpebral conjunctiva. **A**: Integrin β1 (green) and CK4 (Red); **B**: Integrin α6 (green) and CK4 (red); **C**: Talin1 (green); **D**: Vinculin (green); **E**: Profilin1 (green); **F**: Double staining of profilin1 (red) and phalloidin (green); **G**: Paxillin (green); **H**: Double staining of paxillin (red) and phalloidin (green); **I**: FAK (green); **J**: Double staining of FAK (red) and phalloidin (green); **K**: Cofilin1 (green); **L**: Double staining of cofilin1 (red) and phalloidin (green); **M**: Gelsolin (green color); **N**: Double staining of gelsolin (red) and phalloidin (green); **P**, **Q**: Integrin β1 (green); **R**, **S**: Paxillin (green); **T**, **U**: Profilin1 (green). Blue color is DAPI as a counterstain, staining nuclear.

**Figure 2:**
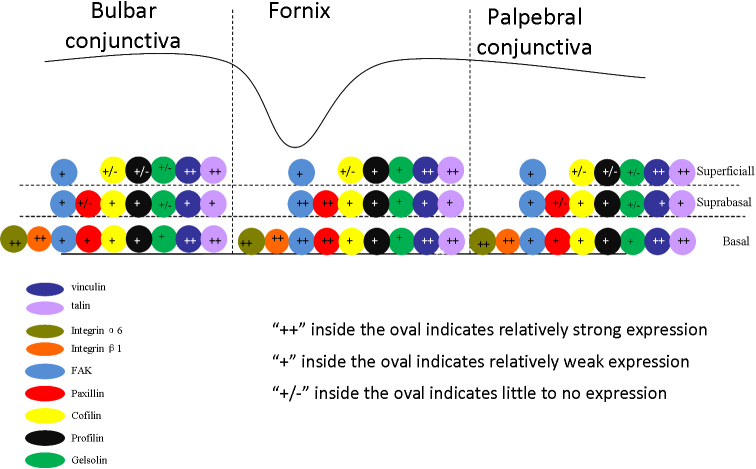
Summary of the microfilament regulators distribution in the forniceal, palpebral and bulbar conjunctival epithelia.

In this study, integrin β1 (Figure 1A) and integrin α6 (Figure 1B) were strongly expressed in the basal layer of the forniceal, bulbar, and palpebral conjunctivae. The expression of talin (Figure 1C) and vinculin (Figure 1D) were both seen in the full layer of the forniceal, bulbar, and palpebral conjunctivae, with the greatest intensity of expression seen in the basal and superficial layers. Profilin1 was moderately expressed in the full layers of forniceal, palpebral and bulbar conjunctivae (Figure 1E,F). Paxillin was expressed with the greatest intensity in the forniceal conjunctivae; it appeared with diminishing intensity along the basal layer of the palpebral and bulbar conjunctivae, and it ended at the epithelium of the mucocutaneous junction (Figure 1G,H). FAK was expressed in all layers of the palpebral, forniceal, and bulbar conjunctivae; however, the intensity was greater in the fornix (Figure 1I,J). Cofilin1 was weakly expressed in all layers of the forniceal, palpebral and bulbar conjunctivae (Figure 1K,L). Gelsolin weakly located in the full layers of forniceal, palpebral, and bulbar conjunctivae (Figure 1M,N). Figure 2 shows the summary of the distribution of the microfilament regulators in the conjunctival epithelium at forniceal, palpebral and bulbar sites.

The expression pattern of integrin β1 (Figure 1P,Q), paxillin (Figure 1R,S), and profilin1 (Figure 1T,U) in the bulbar conjunctivae most closely resemble its immediate adjacent epithelium at the limbus.

Female and male mice did not demonstrate differences in the expression pattern of microfilament regulators at conjunctival forniceal, bulbar, or palpebral sites.

### Gene expression of microfilament regulators in conjunctival forniceal, bulbar, and palpebral epithelia

**Figure 3:**
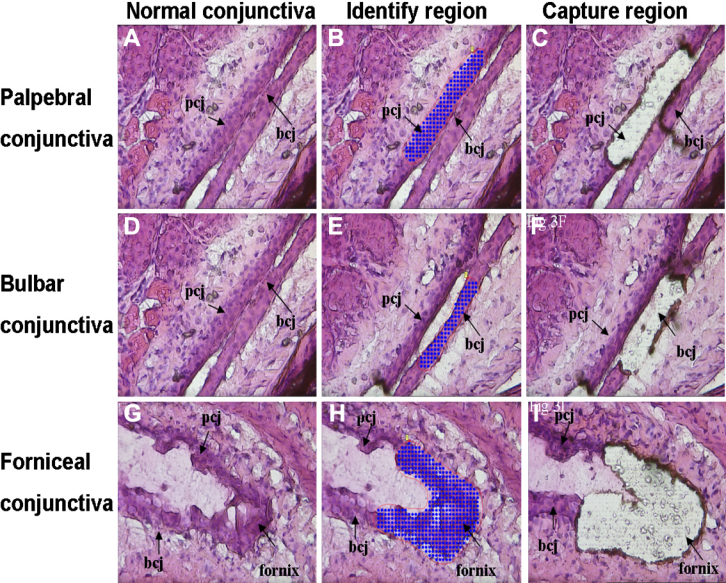
PALM laser dissection. A, **D**, **G**: OCT-embedded mouse eye tissue was cut at 10 μm, fixed and stained with hematoxylin and Eosin. **B**, **E**, **H**: Palpebral, bulbar and forniceal conjunctival epithelium region were identified and circled. **C**, **F**, **I**: The selected region was cut from the surrounding cells, and the same region was captured leaving a clear margin of surrounding cells. All pictures were taken at 400×. bcj, bulbar conjunctiva; pcj, palpebral conjunctiva.

For further analysis, it was necessary to localize the epithelial cells from the various regions to determine if the cellular analysis corroborated the immunohistochemical findings. The PALM CombiSystem made it feasible to separate the conjunctival epithelia from the underlying fibrous tissue (Figure 3).

**Figure 4:**
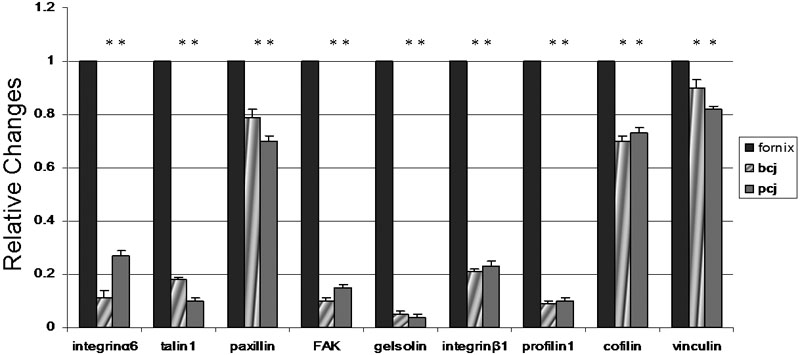
Relative real-time PCR results. Fold difference of each target gene expression among different samples in comparison with conjunctival forniceal epithelial cells. The calculation of the fold difference was described in Methods. The asterisk indicates a significant difference, p<0.05, compared to the transcript level in forniceal epithelial cells. bcj, bulbar conjunctiva; pcj, palpebral conjunctiva.

Quantitative RT–PCR was used to determine the relative abundance of each target transcript in the conjunctival forniceal, bulbar, and palpebral epithelia, which was removed by PALM CombiSystem laser dissection (n=60 for each conjunctival region). The normalized expression levels of each target transcript in the conjunctival bulbar and palpebral epithelia were not significantly different (p>0.05 and n=60 for each conjunctival region); however, both yielded levels that were lower than those found in the forniceal conjunctival epithelia (p<0.05 and n=60 for each conjunctival region). A bar graph that summarizes the fold differences of each target transcript in the conjunctival bulbar and palpebral epithelia as compared with the forniceal epithelia is shown in Figure 4. There were no significant differences between female and male mice.

### Western blot analysis

**Figure 5:**
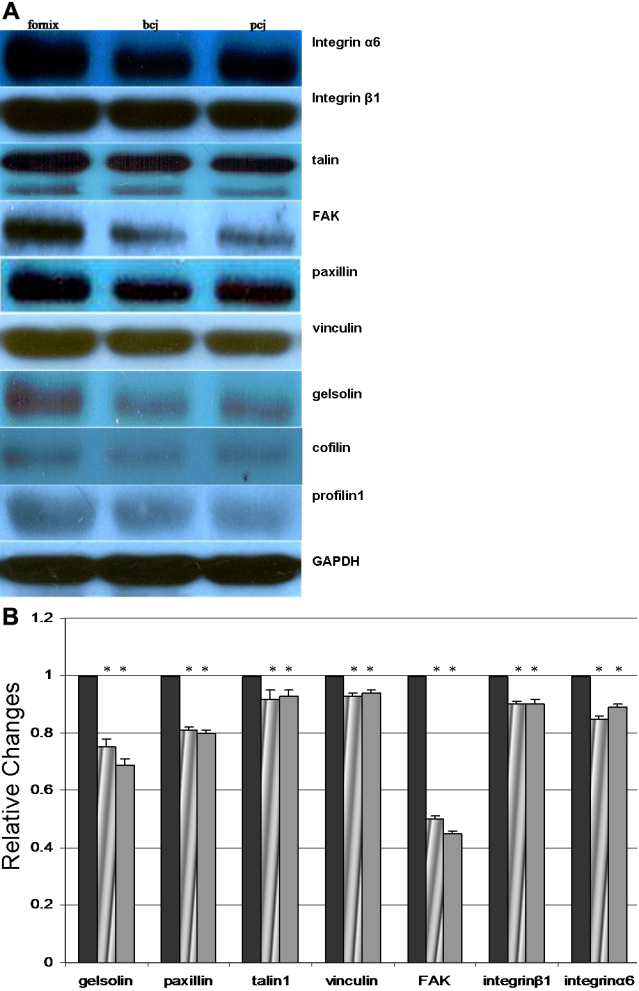
Western blot analysis. GAPDH was used as the loading control. **A**: The proteins identities are indicated on the right. **B**: Band intensity was quantified by densitometry and the fold difference of each microfilament regulator in the conjunctival bulbar and palpebral epithelia compared to the conjunctival forniceal epithelia was expressed graphically. bcj, bulbar conjunctiva; pcj, palpebral conjunctiva.

Western blot analysis was performed to determine the relative levels of protein expression in the conjunctival forniceal, bulbar, and palpebral epithelia. Laser dissection with the PALM CombiSystem was used to separate the conjunctival epithelia from the underlying fibrous tissue (Figure 3). The intensity of the bands for the microfilament regulators was relatively high in the conjunctival fornix as compared with the bulbar and palpebral epithelia (p<0.05 and n=60 for each conjunctival region; Figure 5A,B). Similar levels of microfilament regulators were observed in both conjunctival bulbar and palpebral epithelia (p>0.05 and n=60 for each conjunctival region). A bar graph that summarizes the fold differences of each microfilament regulator in the conjunctival bulbar and palpebral epithelia as compared with the conjunctival forniceal epithelia is shown in Figure 5B.

## Discussion

After a review of the existing literature regarding the expression of microfilament regulators in the conjunctiva, it was found that only a few reports about the conjunctiva or the cornea were available. Antibodies to talin and vinculin did not react with the normal rabbit corneal epithelial cells [21] and FAK labeling was not seen in the mouse corneal epithelium [22]; however, in the present study, they were readily observable in mouse conjunctival epithelium (Figure 1C,D,I,J). In situ hybridization has shown that the mouse corneal epithelium contains relatively little gelsolin [23]. Similarly, in the present study, it was found that gelsolin had a weak presence in the conjunctival epithelium (Figure 1M,N). Currently, there is no information about the expression of profilin1 and paxillin in the cornea or the conjunctiva.

Because the conjunctiva epithelium has formed the basis for regenerative stem-cell transplants, the localization of the potential stem cells is of interest [24]. Previous studies have indicated that bulbar conjunctival epithelial cells may have a lineage from the limbus. Zajicek and colleagues [25] proposed that conjunctival and corneal epithelia are the descendants of an uncommitted stem cell that generates two differentiation pathways. Pe'er and colleagues [26] also suggested that an undetermined limbal stem cell generates two epithelial cell lines that lead to the corneal and conjunctival epithelia. In the present study, the expression pattern of integrin β1 (Figure 1P,Q), paxillin (Figure 1R,S), and profilin1 (Figure 1T,U) in the mouse bulbar conjunctival epithelium most closely resembles that of the spatially related limbal epithelium.

This study has shown for the first time that the analysis of microfilament regulators from the EME to the intracellular microfilament system demonstrates expression differences along the forniceal, bulbar, and palpebral conjunctival epithelia. Although forniceal, bulbar, and palpebral conjunctiva exhibited similar morphologic and biochemical features, it is intriguing to note that a subset of microfilament regulators have significant quantitative differences at varied conjunctival sites (Figure 4 and Figure 5). Integrin-mediated focal adhesions serve as the bridge between the EME and the intracellular microfilament system in addition to relaying signals from the EME to the nucleus [27,28]. The EME–microfilament contacts across the integrin are strengthened by the incorporation of microfilament regulators such as talin, paxillin, and FAK into focal adhesion complexes [29]. In this study, the expression levels of FAK, paxillin, and talin were significantly higher at conjunctiva forniceal sites than at palpebral and bulbar sites (Figure 4 and Figure 5). Hence, the integrin-mediated linkages between the EME and the intracellular microfilament are increased by higher amounts of FAK, paxillin, and talin at forniceal sites as compared with palpebral and bulbar sites.

Vinculin potentially serves as a stabilizing protein in the focal adhesion complex; therefore, the amount of vinculin may be indicative of the cell motility on a substrate [30,31]. Increased levels of vinculin promote cell adhesion and reduce cell motility [32] in addition to stabilizing the integrin–cytoskeleton linkage [33]. In the present study, the expression level of vinculin was significantly higher at forniceal sites as compared with palpebral and bulbar sites (Figure 4 and Figure 5). Therefore, the data point to a more mobile state of conjunctival epithelial cells at palpebral and bulbar sites as compared with forniceal sites.

Actin filaments have important functions for stabilizing cell–cell and cell–matrix contacts [34]. Cell motility and crawling are predicated on rapid dynamic actin reorganization [35-37]. Profilin stabilizes actin structures, which are generally dynamic in nature [38]. Actin-filament stability was found to increase in proportion to profilin concentration in Chinese hamster ovary cells [39]. The phenotypes displayed by yeast cells and drosophila nurse cells that were deficient for profilin were consistent with the ability of profilin to stabilize actin filaments [40,41]. Kudryashov and colleagues [42] suggested that cofilin can stabilize alternative longitudinal contacts that substitute for those that have been disrupted or weakened; this is consistent with several studies that have implicated cofilin in the stabilization of actin structures under certain in vitro and in vivo conditions [43,44]. In the present study, the expression levels of cofilin and profilin were significantly higher at conjunctival forniceal sites than at palpebral and bulbar sites (Figure 4 and Figure 5). Thus, the current data suggest that the stability of actin filaments within epithelial cells at conjunctival forniceal sites may be increased as compared to bulbar and palpebral sites.

Investigations of the microanatomic compartments of epithelial stem-cell systems reveal one common feature: stem cells are usually considered to be part of the basal layer of the epithelium. For example, corneal epithelial stem cells are concentrated in the basal limbal region [45-47], interfollicular epidermal stem cells are clustered at the bottom of the deep rete ridges [48,49], and intestinal stem cells have been postulated to reside in the crypt [50]. With respect to the preferential stem-cell location, it has been hypothesized that conjunctival epithelial stem cells reside in the fornix, which is a finger-like invagination that is similar to the intestinal crypt [51,52]. Because epithelial stem cells are usually located along the basement membrane, a high level of expression of certain integrins such as integrin β1 and α6, which mediate cell interactions in the EME, may stabilize the stem cells and help them to maintain their positions in the niche [53-56]. If integrins are involved in the regulation of stem-cell behavior in the epithelia, one might expect to see some differences in the expression levels of the integrins that are present in the forniceal, bulbar, and palpebral conjunctival epithelia. This study has shown just such a difference: the expression levels of integrin β1 and α6 are significantly higher at conjunctival forniceal sites than at palpebral and bulbar sites by RT–PCR and western blot analysis (Figure 4 and Figure 5).

In conclusion, conjunctival epithelial cells have a more stable intracellular interaction between EME and intracellular microfilament in the forniceal conjunctiva compared to epithelial cells in the palpebral or bulbar conjunctiva.
